# Health Connector: Bridging the Gap Between Healthcare and Transportation

**DOI:** 10.1093/geroni/igaf122.1293

**Published:** 2025-12-31

**Authors:** Jenna Kubiak

**Affiliations:** Flexlynqs, Iowa, United States

## Abstract

Access to healthcare remains a significant challenge for many individuals due to transportation barriers. The Health Connector program addresses this by integrating transportation services with healthcare appointment scheduling, creating a seamless mobility solution for all populations. This initiative directly links transit resources with healthcare appointments through a unified platform. To rigorously assess program effectiveness during the ongoing 18-month pilot, Iowa State University analyzed specific performance measures developed during the research phase. Data collection, including rider surveys, trip data analysis, and interviews with healthcare and transit staff provided continuous insights into the program’s impact and effectiveness. Preliminary findings suggested positive trends in patient satisfaction with transportation services. This project continues to highlight the critical importance of robust middleware integration and strong stakeholder communication for successful implementation. Innovative transportation solutions play a vital role in advancing health access, and data from the Health Connector project demonstrates the impact of integrated mobility strategies on healthcare outcomes. Attendees will explore how leveraging technology, stakeholder engagement, and data-driven decision-making can create sustainable, accessible transportation options that improve health outcomes and overall community well-being.

